# Selective and sensitive detection of cinnamaldehyde by nitrogen and sulphur co-doped carbon dots: a detailed systematic study†

**DOI:** 10.1039/c8ra09285k

**Published:** 2018-12-19

**Authors:** Suraj Konar, Dipanjan Samanta, Subhajit Mandal, Subhayan Das, Madhusudan Kr Mahto, Manisha Shaw, Mahitosh Mandal, Amita Pathak

**Affiliations:** Department of Chemistry, Indian Institute of Technology Kharagpur West Bengal 721302 India ami@chem.iitkgp.ernet.in +91-3222-281922 +91-9434-038730; Department of Chemistry, R. D. & D. J. College Munger, Munger University Bihar 811201 India; School of Chemical Sciences, Indian Association for the Cultivation of Science Kolkata West Bengal 700032 India; School of Medical Science and Technology, Indian Institute of Technology Kharagpur West Bengal 721302 India

## Abstract

Nitrogen and sulfur co-doped carbon dots (NSCDs) synthesized through one-pot microwave-assisted pyrolysis of tartaric acid and thioacetamide have been used as a fluorescent probe for the sensitive and selective detection of clinically important organic aldehyde cinnamaldehyde. The as-prepared NSCDs displayed blue fluorescence (∼12% quantum yield), excellent aqueous solubility along with pH and excitation wavelength dependent emission behavior. In comparison to other aldehydes (formaldehyde, acetaldehyde, propionaldehyde, butyraldehyde, valeraldehyde, hexanal, crotonaldehyde and benzaldehyde) the fluorescence intensity of NSCDs was significantly quenched in the presence of cinnamaldehyde and the reduced intensity was linearly proportional to the concentration of cinnamaldehyde in the range of 0–15 mM with a detection limit of 99.0 μM. The fluorescence quenching of NSCDs was mainly attributed to the photo-excited electron transfer between NSCDs and aldehydes which was confirmed by measuring the life-time through time-resolved luminescence spectroscopy, energy levels of NSCDs through cyclic voltammetry (CV) and energy levels of aldehydes by density functional theory (DFT) based analyses. MTT assay of the NSCDs also proved their good biocompatibility and low toxicity towards human fibroblast cells thereby validating their suitability as a biologically relevant fluorescent probe for sensing cinnamaldehyde.

## Abstract 2

Nitrogen and sulfur co-doped carbon dots (NSCDs) have been used as a fluorescent probe for the sensitive and selective detection of clinically important organic aldehyde cinnamaldehyde.

## Graphical abstract

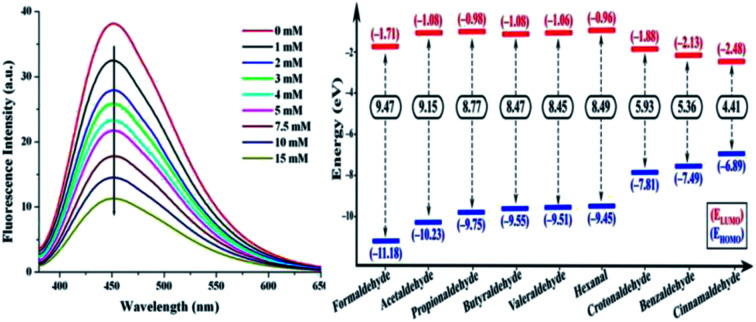

## 1. Introduction

Cinnamaldehyde is an important chemical constituent of the essential oil extracted from cinnamon bark which is frequently used as a stomachic, antipyretic and antiallergic drug.^1^ The spicy taste and fragrance of cinnamon are due to the presence of cinnamaldehyde after the absorption of oxygen.^2^ Cinnamaldehyde helps in lipid peroxidation and peroxynitrite induced nitration process and it has protective effects on the cardiovascular system.^3,4^ It is also well known for showing antityrosinase activity.^5^ It produces hypotensive effects, which are possibly due to peripheral vasodilatation in anesthetized dogs and guinea pigs.^6^ Cinnamaldehyde restricts the progress of hypertension in types 1 and 2 diabetes by abridging vascular contractility; in addition it shows an insulinotropic effect in insulin deficiency.^7^ It is also established that the derivative of cinnamaldehyde, 2′-hydroxycinnamaldehyde, inhibits tumor growth and shows anticancer effect.^8^ Because of its considerable role in clinical science, researchers have proposed a number of analytical techniques for the detection of cinnamaldehyde which include thin-layer chromatography (TLC),^9^ gas chromatography (GC),^10^ high-performance liquid chromatography (HPLC)^1^ and reverse phase HPLC^11^ although they are complicated and time consuming. Cinnamaldehyde finds its application as a flavoring agent, odor agent, and used as a fragrance agent in global market like cosmetics, soaps, detergents, deodorants, shampoos, *etc.* Thus, it is essential to develop a simple, sensitive and selective analytical approach to detect cinnamaldehyde.

Carbon materials like carbon nanotubes, graphene and carbon dots are gaining attention as promising applicants in the fields of sensing,^12–14^ catalysis,^15–17^ solar cells,^18^ light emitting devices,^19^ drug delivery^20^ and bioimaging^21^ purposes. Carbon dots (CDs) have attracted considerable interest for last decade due to their appealing advantages comprising bright photoluminescence, convenient synthesis, excellent biocompatibility, aqueous solubility and chemical inertness.^22–24^ These fascinating physical and chemical properties made them promising applicant in those fields. Overwhelmed by their multimodal applications in diverse fields, various preparative techniques have been developed to synthesize CDs for instance laser-ablation,^25^ electrochemical oxidation,^26^ combustion,^27^ hydrothermal^28^ and microwave-assisted pyrolysis.^29^ Among these techniques, the microwave assisted pyrolysis has gained the growing attention because of its low expenditure, continuous and rapid heating which greatly enhances the reaction efficiency and quantum yield of CDs.^30^ For regulating the electronic and fluorescence properties of CDs researchers has been used doping into CDs with heteroatoms (*e.g.* nitrogen,^31^ boron,^32^ sulphur^33^ and phosphorous^34^) as an efficient approach. Among these dopants, nitrogen doping is the supreme method to enhance their quantum yield and shows multicolor photoluminescence (PL) property by the process of charge transfer in between electron rich nitrogen and electron deficient carbon.^35^ However, the doping of sulfur into CDs would seem to be quite difficult because of their small electronegativity difference and larger size of S atom than C atom.^36^ Thus, S-doped CDs have scantly reported in literature although nitrogen and sulphur co-doped CDs (NSCDs)^37^ have also been synthesized by few research groups for detecting various metal ions^38^ and nanoparticles,^39^ cellular imaging^36^ and photosensitizer.^40^ The sulfur atoms existing in NSCDs were confirmed to be synergistic for doping nitrogen in CDs as reported by Ding *et al.*^41^ They proposed that, C=O groups on the surface of CDs were considered to be main emission centers for blue luminescence, while C=N and C–N bonds in the form of polyaromatic structures were considered to be the key factors for promoting the fluorescence of NSCDs.^41^ Shi and co-workers also reported the strong PL properties of NSCDs arising due to the formation of polyaromatic organic fluorophores.^42^

In this work, we have synthesized nitrogen and sulphur co-doped photoluminescent NSCDs through one-pot microwave assisted pyrolysis of tartaric acid and thioacetamide. The physicochemical and optical properties of these NSCDs were investigated using a series of spectroscopic and microscopic characterization techniques. The synthesized NSCDs showed bright photoluminescence (PL) property and excitation wavelength dependent emission behavior. With the addition of cinnamaldehyde the fluorescence intensity of NSCDs was gradually attenuated. Other than cinnamaldehyde we also observed the minimal change in PL intensity of NSCDs in presence of various aliphatic and aromatic aldehydes such as formaldehyde, acetaldehyde, propionaldehyde, butyraldehyde, valeraldehyde, hexanal, crotonaldehyde and benzaldehyde. Cyclic voltammetry measurement and DFT analyses corroborated that the electrons in lowest unoccupied molecular orbitals (LUMOs) of aldehydes (electron donor) are get transferred to the LUMO of NSCDs (electron acceptor) after excitation. The process of photo-excited electron transfer is energetically more feasible in cinnamaldehyde-NSCDs couple than other aldehyde-NSCDs systems. The cellular uptake study and MTT assay of NSCDs in human fibroblast cells shows the innocuous nature of NSCDs. To the best of our knowledge, it is the first report of employing NSCDs as a fluorescent probe for sensing cinnamaldehyde. The fluorometric detection of cinnamaldehyde shows many virtues including simplicity, rapidity, high sensitivity and excellent selectivity, which qualifies non-toxic NSCDs as an efficient nano biosensor in clinical diagnostic applications.

## 2. Materials and methods

### 2.1 Materials

All of the reagents, tartaric acid (C_4_H_6_O_6_, Merck Ltd, Mumbai, 99.7%), thioacetamide (C_2_H_5_NS, S.D. fine chemical Ltd, India, 99%), dopamine hydrochloride (Sigma-Aldrich, 98%), *trans*-cinnamaldehyde (C_9_H_8_O, Alfa Aesar, 98%), formaldehyde (CH_2_O, Merck, 41% w/v), acetaldehyde (C_2_H_4_O, Alfa Aesar, 98%), propionaldehyde (C_3_H_6_O, Alfa Aesar, 97%), butyraldehyde (C_4_H_8_O, Alfa Aesar, 98%), valeraldehyde (C_5_H_10_O, TCI Chemicals, India, 95%), hexanal (C_6_H_12_O, TCI Chemicals, India, 95%), crotonaldehyde (C_4_H_6_O, Alfa Aesar, 98%) and benzaldehyde (C_7_H_6_O, Alfa Aesar, 99%) were analytically pure and used without further purification. Dulbico's Modified Eagle's Medium with high glucose (DMEM with high glucose, Gibco), PBS (Phosphate-buffered saline, Sigma), paraformaldehyde (Sigma), Rhohamin-Phalloidin (Invitrogen), MTT [3-(4,5-dimethylthiazol-2-yl)-2,5-diphenyltetrazolium bromide, Sigma], DMSO (dimethyl sulfoxide, Sigma), Trypsin–EDTA solution (HiMedia), Fetal bovine serum (FBS, HiMedia). Milli-Q water was used throughout the experiment.

### 2.2 Synthesis of N, S co-doped carbon dots (NSCDs)

In a typical synthetic procedure, tartaric acid and thioacetamide were taken in 1 : 1 mol ratio and added into milli-Q water. The mixture was sonicated to form a homogeneous solution and then heated in a microwave oven (800 W, 3 min). During this process, the mixture changed from colorless liquid to black solid, indicating the formation of carbon dots (NSCDs). The NSCDs in supernatant were retained and purified by dialysis against Milli-Q water using dialysis membrane (MWCO 12 000 Da, Sigma Aldrich) for 24 h and placed for freeze-drying. The freeze-dried sample was used for detailed characterization and detection of cinnamaldehyde.

### 2.3 Characterization of NSCDs

The X-ray diffraction (XRD) analysis of synthesized NSCDs was performed in the range of 2*θ* from 15–80° using Bruker AXS Diffractometer D8 powder XRD (Germany) equipped with Cu-Kα (*λ* = 0.154 nm) as a radiation source at 40 kV and 30 mA power, at a scan rate of 3° min^−1^. The presence of passivating agents in the surface of NSCDs was confirmed by recording the Fourier transformed infrared (FTIR) spectrum within 4000–400 cm^−1^ in a Perkin-Elmer Spectrum RX-II (Model no. 73713, USA) instrument. The morphology of synthesized NSCDs was examined by transmission electron microscope (TEM) in a TECNAI G2 20S-TWIN (Japan) machine with an acceleration voltage of 200 kV. Aqueous solution of NSCDs was drop casted into a 300 mesh carbon coated copper grid and the solvent was evaporated for overnight at room temperature. The Raman spectrum of NSCDs was recorded in a HORIBA JobinYvon T64000 Raman spectrometer (Japan) by using an Ar–Kr laser source of fixed wavelength (*λ* = 514 nm), equipped with a microscope (model BX41 Olympus, Japan). The X-ray photoelectron spectrum (XPS) of NSCDs was recorded in a PHI 5000 Versa probe-II scanning microprobe (United States) outfitted with an Al-Kα X-ray monochromator (1486.7 eV). The binding energy scale of the spectrum has been calibrated by standard value of C 1s at 284.6 eV. The surface charge potential of synthesized NSCDs was measured at different pH values (2 to 10) in a Nano Particle Analyzer HORIBA SZ-100 instrument (Japan). The measurements were repeated for thrice and accepted the average value in order to gain precise results. The optical properties of the samples were analyzed by carrying out the absorption spectrum in UV-vis spectrophotometer (SHIMADZU UV-2450, Japan) and photoluminescence (PL) studies recorded in Fluorescence spectrophotometer (HITACHI F-7000, Japan). The highest occupied molecular orbital (HOMO) and lowest occupied molecular orbital (LUMO) energy levels of the NSCDs was estimated by cyclic voltammogram (CH Instruments, Electrochemical Analyzer) by using a standard three-electrode system, which consists of a glassy carbon disk as the working electrode, a platinum wire as the counter electrode, an Ag/AgCl as the reference electrode and recorded at a sweep rate of 50 mV s^−1^. The NSCDs electrode was made by drop-casting of NSCDs aqueous solution onto the glassy carbon electrode and the solution was evaporated at room temperature for overnight. 0.1 M potassium chloride (KCl) solution was dissolved in Milli-Q water at room temperature, used as electrolytic solution.

### 2.4 Detection of cinnamaldehyde

The freeze-dried NSCDs powder was dissolved in Milli-Q water for preparing 1 mg mL^−1^ stock solution. 1.0 M stock solutions/dispersions of various aliphatic and aromatic aldehydes such as, formaldehyde, acetaldehyde, propanal, *n*-butanal, *n*-pentanal, *n*-hexanal, crotonaldehyde, benzaldehyde and cinnamaldehyde were prepared in Milli-Q water. To evaluate the selectivity of NSCDs, a fixed volume of these aldehydes solution were separately mixed into a fixed volume of NSCDs solution; incubated for two minutes at room temperature and then the fluorescence spectra (in HITACHI F-7000 Fluorescence spectrophotometer, Japan) were recorded for each set of solutions at the excitation wavelength of 360 nm. To assess the detection range, different sets of solutions of fixed volumes were prepared by mixing various amounts of cinnamaldehyde into fixed volume of NSCDs. The series of solutions were incubated for two minutes at room temperature and the fluorescence spectra were recorded from 380 nm to 650 nm at an excitation wavelength of 360 nm. Each sets of solution were prepared and emission spectrum was recorded for thrice.

### 2.5 DFT calculations of various aldehydes

Full Optimization of ground-state geometries of all the previously mentioned aldehyde molecules was done using the density functional theory (DFT) method using B3LYP,^43,44^ ωB97X-D,^45^ PBE0 ^46,47^ and M06-2X^48^ hybrid functional along with the 6-31+g(d)^49^ basis set in the gas phase. Restricted approach is adopted in the computational analyses for the closed shell structures. The stationary points are characterized by vibrational frequency analysis so that the optimized aldehyde geometries are associated with zero imaginary frequency. Molecular orbitals were evaluated through the formatted checkpoint files as implemented in Gaussian09 software package.

### 2.6 Cellular uptake study of NSCDs

Cellular uptake of NSCDs was studied by seeding human fibroblast cells at a density of 5 × 10^3^ on sterile glass cover slips. The cells were then treated with 100 μg mL^−1^ of NSCDs suspended in DMEM (Dulbecco's Modified Eagle's medium) incomplete medium for different durations (6, 24 and 48 h). After incubation, cover slips were washed with ice-cold PBS and the cells were treated with 3.7% paraformaldehyde for 20 min. Then the actin filaments were stained with Rhohamin-Phalloidin (Invitrogen) dye and then the cells were visualized by confocal laser scanning microscope (CLSM, Olympus FluoView 1000).

### 2.7 Cytotoxicity assay

The cytotoxicity of NSCDs were determined through conventional MTT [3-(4,5-dimethylthiazol-2-yl)-2,5-diphenyltetrazolium bromide] assay with slight modification to the protocol.^50^ Human fibroblast cells were seeded in 96-well plate at a density of 7 × 10^3^ cells per well in DMEM medium supplemented with 10% fetal bovine serum. The cells were incubated at 37 °C in a CO_2_ incubator until they became 70% confluence. Then the complete DMEM medium was replaced by incomplete DMEM medium and the cells were treated with different concentrations of the prepared NSCDs ranging from 1 to 100 μg mL^−1^. For comparison, control wells were treated with incomplete DMEM medium only. After 24 and 48 h of incubation, the medium (containing the NSCDs) were discarded, replaced with 100 μL of MTT solution (1 mg mL^−1^) and further incubated for 4 h for the reduction of MTT to formazan crystals by the viable cells. The unreduced MTT solution was then discarded and 100 μL of DMSO (dimethyl sulfoxide) was added to each well of the 96-well plate to dissolve the formazan crystals formed by the viable cells. Finally, the plates were shaken and the absorbance of formazan dye was measured at 550 nm using a microplate reader. The assay was performed in triplicate. The cytotoxic effect in each of the treatments was expressed as percentage of cell viability relative to the untreated control cells (% control) and calculated by following equation:^24^Cell viability (%) = (OD_treated_/OD_controlled_) × 100%where OD_controlled_ and OD_treated_ was the absorbance in the absence and in the presence of NSCDs.

## 3. Results and discussion

### 3.1 Characterization of NSCDs

**Fig. 1:**
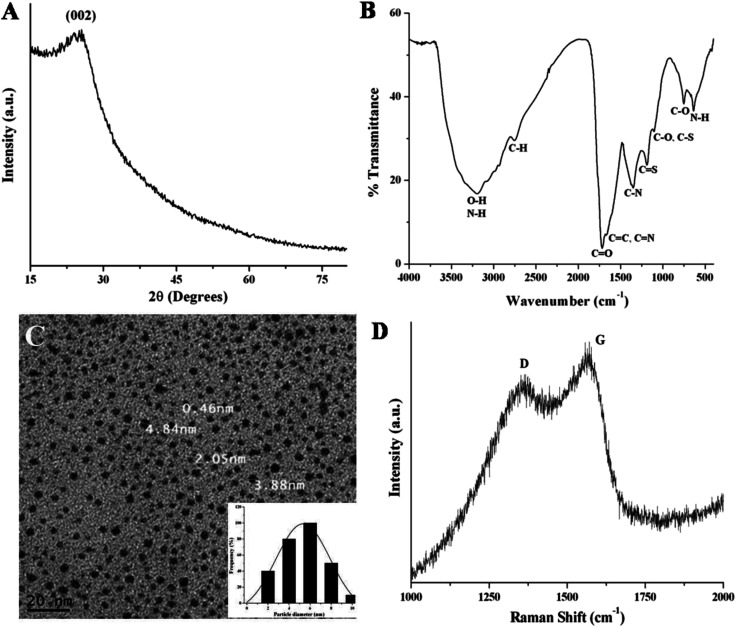
(A) XRD pattern and (B) FTIR spectrum of the NSCDs. (C) TEM image and the corresponding particle size distribution histogram (inset) of NSCDs. (D) Raman spectrum of the NSCDs.

The X-ray diffraction (XRD) pattern is used to investigate the crystallinity of NSCDs. In Fig. 1A, the XRD pattern of NSCDs shows a broad diffraction peak centered at ∼25° (2*θ* value) corresponding to an interlayer spacing of ∼3.55 Å. The value is slightly higher than the interlayer spacing of (002) planes in bulk graphite (3.34 Å), which is probably due to the incorporation of nitrogen, sulfur and oxygen containing groups in NSCDs.^36^

To investigate the surface functional groups in NSCDs, the FTIR analysis was performed and the spectrum depicted in Fig. 1B. In figure, the wide band positioned at ∼3198 cm^−1^ is assigned to the characteristic stretching vibrations of O–H bond while the sharp peak at ∼638 cm^−1^ is ascribed to the out of plane bending of N–H bonds. Another small band at ∼2757 cm^−1^ corresponds to the stretching frequency of C–H bonds. The presence of sharp peaks at ∼1720 cm^−1^ and ∼752 cm^−1^ are assigned to the stretching vibration of C=O groups and in plane bending of C–O bonds respectively. Two distinct peaks positioned at ∼1105 cm^−1^ and ∼1188 cm^−1^ correspond to the stretching vibrations of C–N or C–S bonds and C=S bonds respectively, which confirms that carbon dot is passivated by N and S heteroatoms. Further, the sharp peak found at ∼1351 cm^−1^ is ascribed to the stretching vibration of C–N bond and another small peak at ∼1650 cm^−1^ is assigned to the stretching vibration of C=C groups. The FTIR results indicate the presence of –COOH, –OH and –NH_2_ functional groups on the surface of NSCDs.

The TEM image of NSCDs as shown in Fig. 1C indicates the particles are nearly spherical in morphology and well dispersed. The corresponding histogram as shown in Fig. 1C inset designates that the particle size distribution of NSCDs is in the range of 2–10 nm and the mean particle diameter is found to be approximately 5.32 nm.

The Raman spectrum of NSCDs as shown in Fig. 1D provides the information regarding the crystallinity of carbon dots. Two peaks at 1357 and 1562 cm^−1^ were observed in the spectrum, attributed to D band (disorder) and G band (graphitic) respectively. The D band is related to the A_1g_ breathing mode of vibrations of sp^3^ hybridized carbon atoms in the termination plane of graphite while the G band corroborates the E_2g_ phonon vibrations of sp^2^ hybridized graphitic carbon atoms in a two dimensional hexagonal lattice.^51^ The intensity ratio of the D and G bands (*I*_D_/*I*_G_) is measured to be ∼0.82 which indicates the formation of partially disordered graphitic nature of NSCDs.

**Fig. 2:**
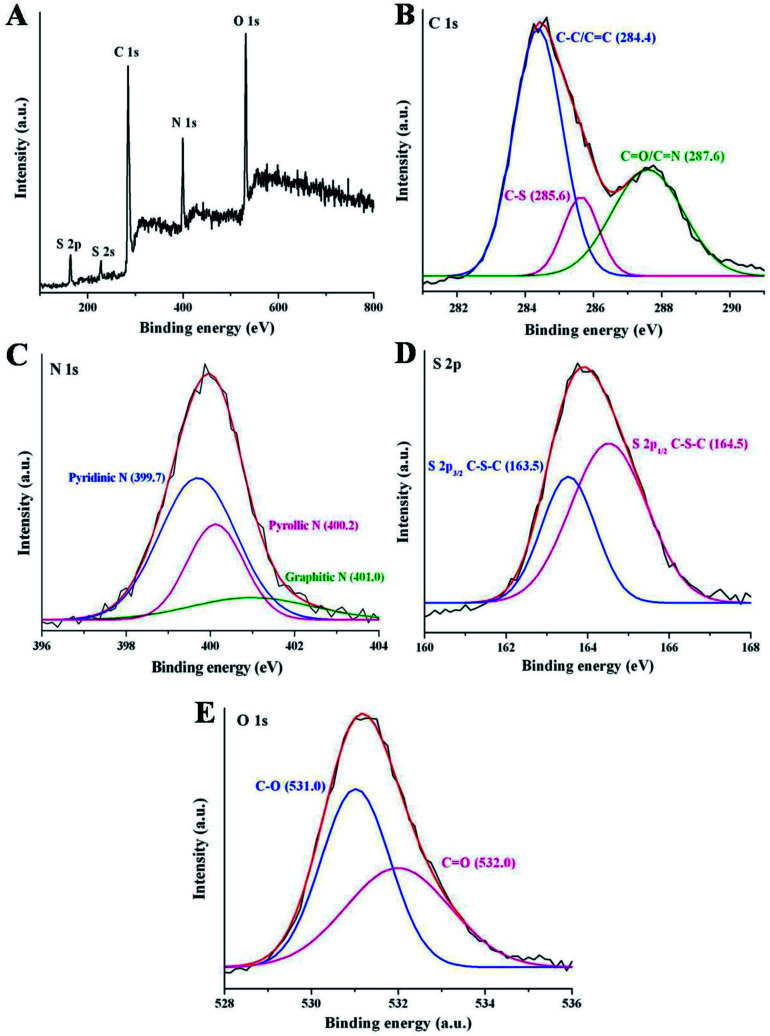
(A) XPS full survey of NSCDs. The high resolution spectra of (B) C 1s, (C) N 1s, (D) S 2p and (E) O 1s of NSCDs.

To further study the surface states and elemental composition of synthesized NSCDs, the XPS study was performed. The XPS survey spectrum of NSCDs is shown in Fig. 2A where five characteristic peaks are observed at around 164 eV, 227 eV, 284.5 eV, 400 eV and 531.2 eV corresponding to S 2p, S 2s, C 1s, N 1s and O 1s, respectively. The relative atomic percentages of C, O, N and S are found to be 46.24, 29.38, 16.47 and 7.91 respectively which indicates that the prepared carbon dot contains nitrogen and sulfur elements definitely. The high-resolution spectrum of C 1s (Fig. 2B) shows three main binding energy peaks at 284.4 eV, 285.6 eV and 287.6 eV, correspond to C–C/C=C, C–S/C–N/C–O and C=O/C=N groups,^36^ respectively. The presence of C=C groups in NSCDs confirm the existence of graphitic nature in their structure. The high-resolution N 1s spectrum (Fig. 2C) has three peaks located at 399.7 eV, 400.2 eV and 401.0 eV, attributed to the pyridinic N, pyrrolic N and graphitic N atoms,^41^ respectively. In the high-resolution spectrum of S 2p (Fig. 2D) there are two major peaks at 163.5 eV and 164.5 eV corresponding to S 2p_3/2_ of –COSH and S 2p_1/2_ of –C–S covalent bond^41^ of thiophene/thiazine S.^36^ The distinct peak at 227 eV of S 2s, is characteristic of the thiol groups in NSCDs.^52^ These observations confirm that various polyaromatic structures are generated during microwave treatment and heteroatoms are successfully doped onto the surface of NSCDs. Deconvoluted spectra of N 1s and S 2p clearly indicate that maximum number of N atoms are doped into the core of the carbon dots aspyridine/pyrrole-like N, whereas most of the S atoms remains as the surface functional groups (–COSH and –SH); only few number of S atoms are doped as thiophene-like S into the carbon core. It suggests that, incorporation of sulfur atoms into an aromatic system compared to incorporation of N requires harsh conditions.^53^ In addition, the high-resolved spectrum of O 1s displays mainly two binding energy peaks positioned at 531.0 eV and 532.0 eV, attributed to the C–O and C=O bonds,^54^ respectively (Fig. 2E). The XPS result of NSCDs is well consistent with the information acquired from the FTIR analysis.

**Fig. 3:**
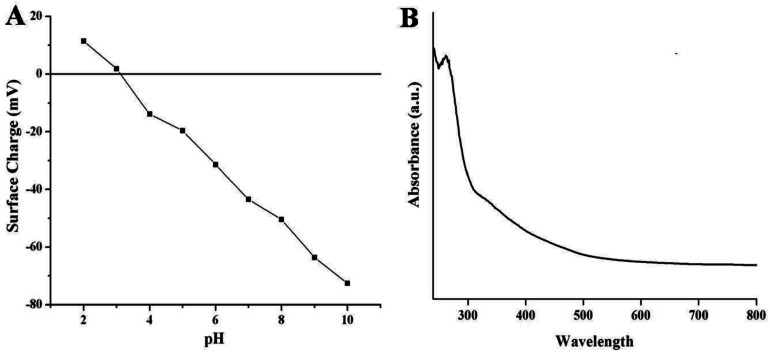
Variation of surface charge of NSCDs at different values of pH (A) and the UV-visible spectra of NSCDs (B).

Fig. 3A represents the surface charge potentials of NSCDs at different pH values. The isoelectric point (IEP) of NSCDs is found to be 3.1, indicating abundance of negatively charged –COOH and –OH groups in their surface. The high value of zeta potential (−43.5 mV at pH = 7) helps to restrict the precipitation or flocculation of NSCDs as a result of intermolecular electrostatic repulsion, offering high solubility in aqueous medium.

The UV-vis absorption and fluorescence spectra of NSCDs solution were recorded to study the optical properties of NSCDs. The absorption spectrum of NSCDs is shown in Fig. 3B where two broad and weak absorption bands are observed in the region of 250–270 nm and 320–350 nm. The band at 250–270 nm is attributed to the π → π* transition of the conjugated C=C bonds originated from the carbon core whereas the band at 320–350 nm is ascribed to the n → π* transition of C=O and C=N bonds respectively.^38^ The broad band at 350 nm may be observed due to the presence of aromatic π-orbitals in carbon dots.^55^

**Fig. 4:**
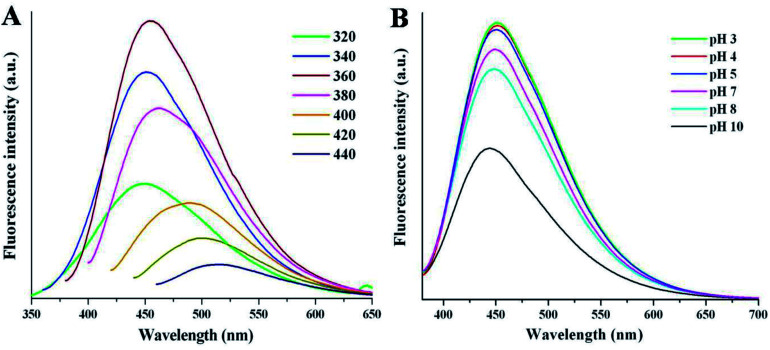
The fluorescence spectra of NSCDs at (A) various excitation wavelengths and (B) different pH values at *λ*_ex_ = 360 nm.

The fluorescence emission spectrum is one of the most fascinating features of carbon dots from an application viewpoint. Fig. 4A shows the emission spectra of NSCDs at different excitation wavelengths (*λ*_ex_). The spectrum shows that the *λ*_em_ peaks remains almost unaltered at 449–460 nm when the *λ*_ex_ is varied from 320 to 380 nm; however, the emission peaks are red-shifted from 485 to 515 nm when *λ*_ex_ moves from 400 to 440 nm. The *λ*_ex_-independent emission spectra are probably attributed to the π → π* transitions of the graphitic structure of the carbon cores whereas the *λ*_ex_-dependent PL spectra are derived from the n→ π* transitions (surface states) of the surface-attached functionalities (C=O/C–N/C–S).^56^ The emission spectra are bathochromically shifted with the increase in *λ*_ex_ which indicates that the emission maxima can be tuned by adjusting *λ*_ex_ and this phenomenon is very useful for multicolor imaging applications.^57–60^ The emission study shows that NSCDs emits blue color and it may be attributed to the presence of C=O groups on the surface of CDs and C=N/C–N bonds decorated in the core of CDs.^41^ The quantum yield of synthesized NSCDs is measured to be ∼12% using quinine sulfate as a standard (Fig. S1, ESI†).

Apart from the excitation-dependent emission behaviour of NSCDs, the emission behavior of the NSCDs was also investigated at different pH values (from pH 3 to 10) as shown in Fig. 4B. We noticed that the emission spectra of the NSCDs are quite unchanged in acidic and neutral range of pH although the fluorescence intensity of NSCDs decreases pronouncedly by ∼50% in strongly alkaline condition (pH 10). This observation implies the fluorescent species located in the NSCDs should have acidic sites that are corresponding to the blue emission.^61^ We also observed that NSCDs exhibit excellent photostability over a period of three months. The excellent stability in acidic pH and photostability of NSCDs makes it promising candidate for a new class of fluorescent probes which may be used as a pH sensor, biosensor and bio-marker.

Furthermore, the electron accepting and donating capability of synthesized NSCDs was investigated by monitoring the emission behavior of NSCDs in presence of electron donor (dopamine in acidic aqueous solution) and electron acceptor (dopamine under alkaline conditions) at *λ*_ex_ = 360 nm.^62^ The fluorescence intensity of NSCDs is quenched by the addition of dopamine in acidic as well as alkaline conditions. The observed Stern–Volmer quenching constant (*K*_SV_) of NSCDs and dopamine system in acidic and alkaline conditions are calculated as 83.33 M^−1^ and 58.33 M^−1^, respectively (data not shown). The above results established that NSCDs can be used as either an electron donor or an acceptor, although the electron accepting ability of NSCDs is more than the electron donating capability.

### 3.2 Sensing of cinnamaldehyde by NSCDs

**Fig. 5:**
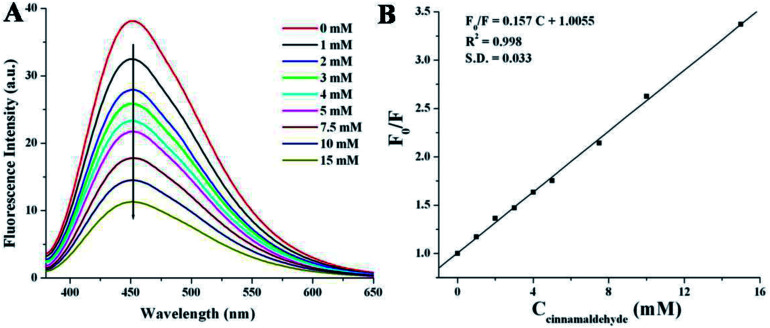
(A) Fluorescence spectra of NSCDs in presence of cinnamaldehyde with various concentrations of 0–15 mM at *λ*_ex_ = 360 nm. (B) The Stern–Volmer plot for NSCDs-cinnamaldehyde system by the emission at 450 nm.

Cinnamaldehyde is an essential compound used in clinical science as anticancer agent, stomachic, antipyretic and antiallergic drug. The researchers have tried to detect cinnamaldehyde by various chromatographic techniques^9,59^ but those methods are time consuming as well as the results suffered from weak accuracy or low selectivity.^9,63^ Herein, we have employed a very simple and rapid optical detection method to monitor the detection of cinnamaldehyde by a fluorescent probe NSCDs in aqueous solution. Fig. 5A shows the emission spectra of NSCDs solutions before and after the successive addition of cinnamaldehyde at the excitation wavelength of 360 nm. The maximum fluorescence intensity of NSCDs gets quenched gradually with the consecutive addition of cinnamaldehyde and the quenching efficiency of NSCDs can finely be fitted with Stern–Volmer equation,^64,65^

1 *F*_0_/*F* = 1 + *K*_SV_[Q]

where, *F*_0_ and *F* are the fluorescence intensities of NSCDs in the absence and presence of cinnamaldehyde at *λ*^max^_ex_ = 450 nm, *K*_SV_ is the Stern–Volmer quenching constant, [Q] is the concentration of quencher *i.e.*, cinnamaldehyde (0–15 mM). The calibration curve is shown in Fig. 5B where we observed a good linear correlation in between *F*_0_/*F* and the concentrations of cinnamaldehyde. The *K*_SV_ value is calculated to be 1.57 × 10^2^ M^−1^ with a correlation coefficient (*R*_2_) value of 0.998. The limit of detection (LOD) value is estimated to be 99.0 μM by using 3*σ* criterion (where, *σ* is the standard deviation of the graph at a signal to noise ratio of three).^66^ The LOD value confirms that NSCDs based fluorescence quenching method explores a better sensing approach for the detection of cinnamaldehyde through less complicated along with less time-consuming fluorescence spectroscopy.

**Fig. 6:**
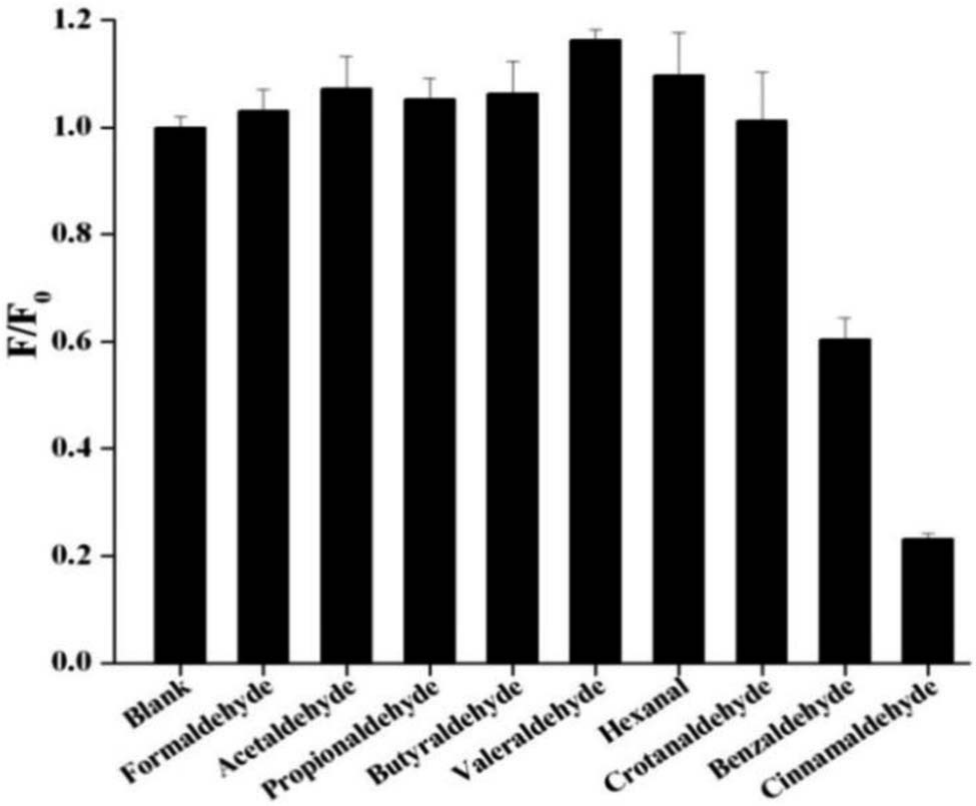
Comparison of fluorescence intensities of NSCDs after the addition of different aldehydes of same concentration.

In order to check the effect of other aldehydes on the sensing of cinnamaldehyde, the effect of maximum emission intensity of NSCDs in presence of various aldehydes is inspected at *λ*_ex_ = 360 nm under the same experimental conditions. Fig. 6 shows the bar diagram of *F*/*F*_0_ (*F*_0_ and *F* are the fluorescence intensities of NSCDs in absence and presence of cinnamaldehyde at *λ*^max^_em_ = 450 nm) *versus* various aldehydes where the concentrations of NSCDs and aldehydes are kept same. We observed that except cinnamaldehyde most of the aldehydes do not induce significant decrease in fluorescence intensity of NSCDs; however benzaldehyde reduces the fluorescence intensity of NSCDs to some extent. Benzaldehyde is about three times less sensible than cinnamaldehyde for quenching the fluorescence intensity of NSCDs which makes NSCDs highly selective to detect the cinnamaldehyde.

### 3.3 Possible mechanism of fluorescence response of the NSCDs to aldehydes

Several kinds of molecular interactions of the quencher molecule with the fluorophore can reduce the fluorescence intensity of fluorescent probe, such as electron or energy transfer, excited-state reaction, and ground-state complex formation.^67^ The quenching mechanisms are usually classified into two categories – dynamic quenching and static quenching. These two types of quenching mechanisms could be distinguished by temperature dependent fluorescence quenching study, fluorescence lifetime measurement and variation in UV-vis absorption spectra.^64^

**Fig. 7:**
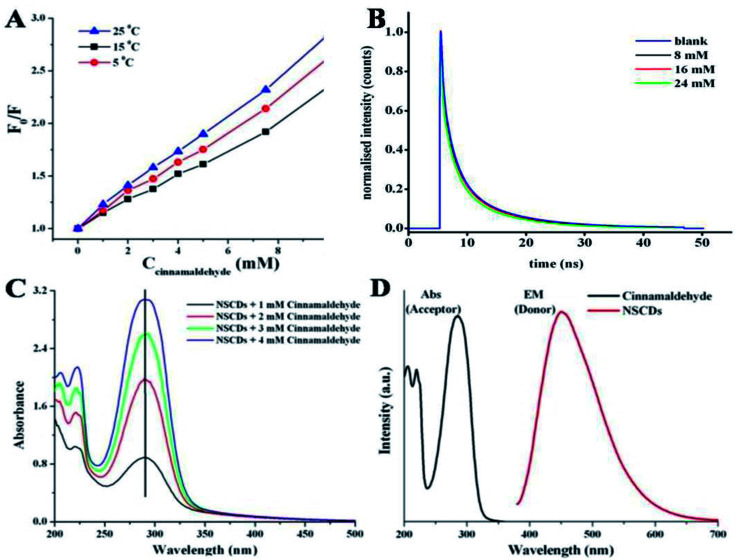
(A) Stern–Volmer plot of NSCDs-cinnamaldehyde system at three different temperature, (B) fluorescence lifetime decays of the acceptor NSCDs in the absence and presence of different concentrations of donor cinnamaldehyde (8 mM, 16 mM, 24 mM), (C) UV-visible absorption spectra of NSCDs in presence of cinnamaldehyde of different concentrations (1–4 mM) and (D) spectral overlap of absorption spectrum of cinnamaldehyde (acceptor) and PL emission spectrum of NSCDs (donor).

The linear Stern–Volmer plot (as shown in Fig. 5B) indicates that quenching mechanism is either static or dynamic.^68^ In static quenching, the quencher binds with the sensor in the ground state forming a non-fluorescent or less fluorescent complex than the fluorophore. The stability of the complex decreases at higher temperature and thus the static quenching constant becomes less.^64^ On the other hand, for dynamic quenching the diffusion-controlled collision is observed in between excited fluorophore and the quencher. The dynamic quenching constant is going to be increased with increasing temperature due to larger number of diffusion controlled collisions between fluorophores and quencher at higher temperature.^64^ To elucidate the mechanism of fluorescence quenching, the temperature dependent quenching experiment was performed at three different temperatures, *viz.* 288, 298 and 308 K. Fig. 7A shows that the slopes of the lines are increasing with increase in temperature, signifying the occurrence of dynamic quenching rather than static quenching. Furthermore, static and dynamic processes can be differentiated by measuring the time resolved fluorescence decay measurements of the probe in presence and absence of the analyte.^69^ In case of static quenching, the non-fluorescent complex is formed in ground state and the unbound fluorophores exhibit their inherent lifetimes. If the quencher molecules are not bound to the fluorophore molecules, the diffusion-controlled dynamic quenching will come into play as an additional non-radiative relaxation pathway and the lifetime of the fluorophore will become shorter. To confirm the dynamic quenching process between NSCDs and cinnamaldehyde, we also recorded the fluorescence decay kinetics of NSCDs in the presence of different concentrations of cinnamaldehyde (8 mM, 16 mM and 24 mM) in aqueous solution by means of time-correlated single-photon counting (TCSPC) measurements. As shown in Fig. 7B, the average fluorescence lifetimes of NSCDs (*τ*_D_) are gradually decreased with increase in concentration of the solutions of cinnamaldehyde (*τ*_D_–A), which suggests that presence of dynamic quenching mechanism operating in this event. The obtained data are fitted by deconvolution as shown in Table S1.† In the absence and presence of 8 Mm, 16 mM, 24 mM cinnamaldehyde the average lifetime values are calculated to be 2.44, 2.36, 2.19 and 1.81 ns respectively. To gain further insight into the fluorescence quenching mechanism, we have investigated the absorption behavior of NSCDs in presence of cinnamaldehyde at different concentrations. In static quenching the absorbance of quencher is supposed to be changed due to the formation of ground state complex with the fluorophore.^70^ As shown in Fig. 7C the absorption maxima are unchanged with the addition of cinnamaldehyde (2–8 μM) in NSCDs solution. This observation also suggests that the quenching mechanism to be dynamic in nature due to the fact that the dynamic quenching only affects the excited state of quencher without any change on the absorption spectrum of quenching molecule.

Dynamic quenching is typically attributed to either an energy transfer or an electron transfer phenomenon.^71,72^ Energy transfer is often considered to be the predominant quenching mechanism at the surface of nanostructures (*e.g.*, Förster resonance energy transfer, FRET). One requirement for an FRET quenching mechanism is that the emission spectrum of the donor fluorophore must overlap with the absorption spectrum of the acceptor.^71^Fig. 7D shows that there is no considerable overlap in between emission spectrum of donor NSCDs and the absorption of the acceptor cinnamaldehyde, which suggests that the quenching of NSCDs by cinnamaldehyde cannot be explained by energy transfer mechanism. Since cinnamaldehyde is a π-electron rich molecule, it is generally believed to be capable of donate electrons to fluorophore through quenching the fluorescence intensity of NSCDs.^73^ For behaving cinnamaldehyde as an electron donor the NSCDs should be served as electron acceptor. We already observed that NSCDs contains acidic sites in pH dependent emission study and the electron accepting ability of NSCDs is favourable in presence of electron donor dopamine in acidic solution. Therefore it is speculated that the electron transfer in between NSCDs (acceptor) and cinnamaldehyde (donor) is the dominant mechanism of luminescence attenuation.

Further, to substantiate the electron transfer in between NSCDs and cinnamaldehyde, their highest occupied molecular orbital (HOMO) and lowest unoccupied molecular orbital (LUMO) energy levels were calculated from cyclic voltammogram (CV) and DFT analyses respectively. The energy of HOMO (*E*_HOMO_) and LUMO (*E*_LUMO_) of NSCDs could be estimated according to the empirical formula:^71^

2 *E*_BG_ = *E*_LUMO_ − *E*_HOMO_

3 *E*_BG_ = *hc*/*λ* = 1240/*λ* (in eV)

*E*_BG_ is the band gap of NSCDs calculated from the absorption edge in the absorption spectrum of NSCDs. *E*_BG_ was calculated to be 2.4 eV. In the anodic sweep of the voltammogram curve, no distinct oxidation peak was observed as shown in Fig. S2,† but in the reverse sweep, a broad reduction peak is observed at a potential (*E*_Red_) of −0.85 V. The potential of reference electrode (Ag/Ag+) is +0.197 V (*vs.* NHE).^74^ The reduction potential (*vs.* NHE) corresponds to the conduction band (CB) or LUMO of the NSCDs. Therefore, the reduction potential of NSCDs (*vs.* NHE) will be: *E*_Red_ = (−0.85 + 0.197) V = −0.635 V. The value of −0.635 V (*vs.* NHE) in eV (*vs.* vacuum) is given by:^18^ −4.5 eV (*i.e.* 0 V *vs.* NHE) – (−0.635 V) = −3.847 ≈ −3.847 eV. The LUMO of NSCDs is therefore fixed at −3.847 eV. Thus the *E*_HOMO_ of NSCDs is calculated to be: (−3.847–2.4) eV = −6.247 eV (*vs.* vacuum).

**Fig. 8:**
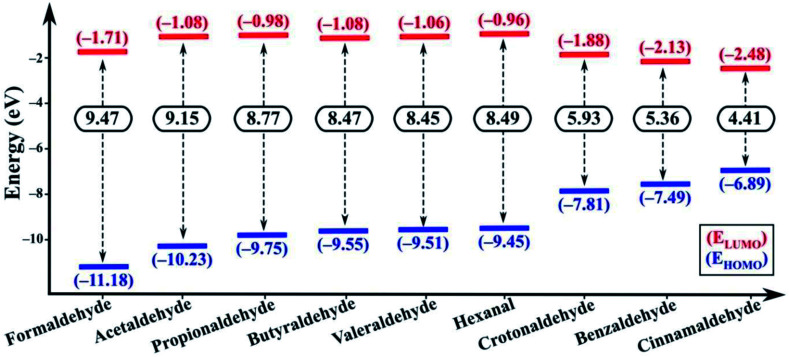
The HOMO and LUMO energy levels of all the aldehydes. The energy levels are in eV.

In the excited state the electron transfer mainly occurs through two pathways. Firstly, upon excitation, the electron in the HOMO of the fluorophore (NSCDs) absorbs a photon and jumps to the LUMO of the fluorophore. In absence of quencher, this electron comes back to the ground state with emission of radiation which is observed as strong fluorescence. In presence of quencher, effective excited state electron transfer from the LUMO of the fluorophore to the LUMO of the quencher is occurred which then comes back to the ground state *via* a non-radiative emission resulting of quenching of fluorescence.^69^ Secondly, during excitation of quencher one electron in HOMO gets transferred to the LUMO of the quencher and then the excited electron induces shifting of electron to the LUMO of the acceptor which then comes back to the ground state *via* a non-radiative emission and consequences quenching of fluorescence.^69^ These two pathways can be differentiated by the possible electron transfer in between LUMO of fluorophore/quencher and LUMO of quencher/fluorophore with respect to their energy levels. If the energy of the LUMO of fluorophore is higher than the energy of the LUMO of quencher the first pathway should be responsible for electron transfer. Whereas, when the energy of LUMO of the quencher is higher than the energy of the LUMO of fluorophore the second pathway results the fluorescence quenching. Both the DFT calculations and CV measurement showed that in our study the second pathway was observed as the energies of LUMO of aldehydes are higher than the energy of LUMO of NSCDs (−3.847 eV) as shown in Fig. 8. Thus, aldehydes are acknowledged as electron donor and NSCDs act as electron acceptor in this study.

The electron-withdrawing substituent in a molecule increase the band gap, while the substitutions with an electron-donating group decrease the band gap (E_g_).^75^ In our study, cinnamaldehyde shows smaller band gap which confirms their higher electron donating ability than other aldehydes. Smaller band gap values of the aldehydes suggest greater ease of electron transfer from the HOMO of aldehydes to LUMO of aldehydes during excitation. E_g_ value is least in case of cinnamaldehyde favoring the electron transfer pathway, while the E_g_ value gradually increases in benzaldehyde and becomes larger in non-conjugated aliphatic aldehydes. Further the LUMO of cinnamaldehyde is closest to the LUMO of NSCDs, which also indicates easy electron transfer from the LUMO of cinnamaldehyde to LUMO of NSCDs, which will eventually facilitate the easier quenching process in case of cinnamaldehyde with NSCDs.

#### 3.3.1 Calculation of the free-energy change in PET

To realize the feasibility of the photo-induced electron transfer (PET) process from aldehydes to NSCDs, the free energy of PET has been calculated using the redox potential of NSCDs and aldehydes. The following Rehm–Weller equation (eqn (4)) has been utilized to calculate the free energy change associated with the PET process.^76,77^

4 Δ*G*_PET_ = *E*^D+/D^ − *E*^A/A−^ − *E*^0,0^

where *E*^D+/D^ is the redox potential of the donor (aldehydes), *E*^A/A−^ is the redox potential of the acceptor (NSCDs), and *E*^0,0^ is the excitation energy of NSCDs. *E*^D+/D^ values were obtained from the DFT calculations. The *E*^0,0^ value for NSCDs was estimated from the point of intersection of the absorption and emission spectra of NSCDs.^76^ The negative Δ*G*_PET_ values (Table 1) for all aldehydes suggest that the process is mainly driven by electron transfer in between NSCDs and aldehydes, although the highest negative value of Δ*G*PET for cinnamaldehyde-NSCDs pair recommends the feasibility of PET from cinnamaldehyde to NSCDs than other aldehydes-NSCDs pairs.

**Table 1:** The values of free-energy change in PET process between NSCDs and aldehydes^a^

Aldehydes	*E* ^D+/D^ (eV)	Δ*G*_PET_ (eV)	Δ*G*_PET_ (kcal mol^−1^)
Formaldehyde	–1.71	–0.88	–20.29
Acetaldehyde	–1.08	–0.25	–5.76
Propionaldehyde	–0.98	–0.15	–3.46
Butyraldehyde	–1.08	–0.25	–5.76
Valeraldehyde	–1.06	–0.23	–5.30
Hexanal	–0.96	–0.13	–3.00
Crotonaldehyde	–1.88	–1.05	–24.21
Benzaldehyde	–2.13	–1.30	–29.98
Cinnamaldehyde	–2.48	–1.65	–38.05

a *E* ^A/A−^ of NSCDs = −3.847 eV ≈ −3.85 eV and *E*^0,0^ = 1240/410 eV = 3.02 eV.

The bimolecular rate constant of luminescence quenching of NSCDs by cinnamaldehyde was calculated using the following equation (eqn (5))^78^

5 *K*_SV_ = *k*_q_*τ*_0_

where *k*_q_ is the bimolecular rate constant of luminescence quenching and *τ*_0_ is the luminescence lifetime of NSCDs in the absence of cinnamaldehyde. The values of the *k*_q_ is calculated to be 4.6 × 10^10^ M^−1^ s^−1^, suggesting that the quenching process is controlled by diffusion of the donor and acceptor in the excited state.^78^

The above result shows that except cinnamaldehyde most of the aldehydes do not induce significant decrease in fluorescence intensity of NSCDs, however benzaldehyde reduces the fluorescence intensity of NSCDs to some extent. In presence of aromatic aldehydes (benzaldehyde and cinnamaldehyde) the π–π stacking interaction plays an important role in between the π-electrons of benzene ring of aromatic aldehydes and the π-electrons of C=C groups present in the core of the NSCDs (confirmed by FTIR and XPS studies), which reduces the distance in between NSCDs and aromatic aldehydes and facilitates the electron-transfer in between NSCDs and aromatic aldehydes efficiently. Although, the sensing probe also response to benzaldehyde but it is about three times less sensible than cinnamaldehyde to get quenched the fluorescence intensity of NSCDs which makes NSCDs highly selective to detect cinnamaldehyde. It is probably due to the presence of extended conjugation in cinnamaldehyde which leads to donate the electrons to NSCDs more than less conjugated benzaldehyde. In summary, all the evidence including the fluorescence lifetime, UV-vis absorption spectra, CV measurement of NSCDs and energy simulation of aldehydes by DFT calculation indicate that NSCDs exhibits good response towards the sensing of cinnamaldehyde through photo-excited electron transfer mechanism.

### 3.4 Cellular uptake and cytotoxicity assay of NSCDs

**Fig. 9:**
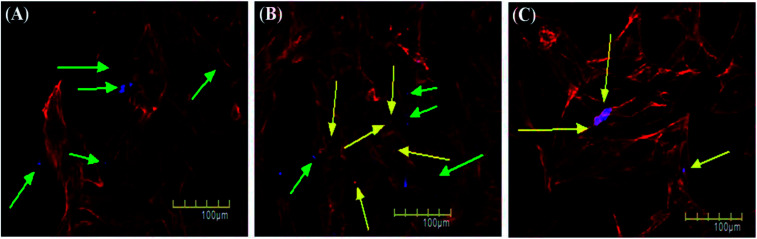
*In vitro* cellular uptake study of NSCDs by fibroblast cells after 6 h (A), 24 h (B) and 48 h (C) of treatment respectively. The NSCDs are blue colored and the actin filaments of fibroblast cells are stained with Rhodamin-Phalloidin (red). The green arrows indicate the NSCDs that are outside the cell and yellow arrows indicate the NSCDs taken up by the cells.

Cellular uptake assay is usually done to assess the capacity of cells to uptake certain particles as well as to determine any toxicity those particles may have.^79,80^ Our NSCDs possess blue fluorescence emission at 450 nm at an excitation of 360 nm, so they could be imaged using the same filter as DAPI (excitation/emission (nm): 358/461). We counter-stained the actin filaments of human dermal fibroblast cells using Rhodamine tagged phalloidin antibodies. Since we used fibroblast for uptake study which is a normal constituent of human tissue we wanted to rule out any long term toxicity NSCDs may have over them. So, we decided to assess the uptake capacity of NSCDs up to 48 hours. We observed that after 24 hours some of the NSCDs were taken up by fibroblasts (Fig. 9b marked by yellow arrow). However, there were still some NSCDs outside (Fig. 9b marked by green arrow). But, after 48 hours most of the NSCDs were taken up by the fibroblasts without any significant alteration of their morphology (Fig. 9c marked by yellow arrow). We can assume from this experiment that the NSCDs are non-toxic to the fibroblasts and are definitely taken up by the cells. However, the uptake is definitely to the slower side compared to some previous studies.^81,82^ One marked difference is that here we used a slower growing non-cancerous cell (human fibroblast) which has significantly slower growth rate compared to cancerous cell lines such as MDA MB 231.

**Fig. 10:**
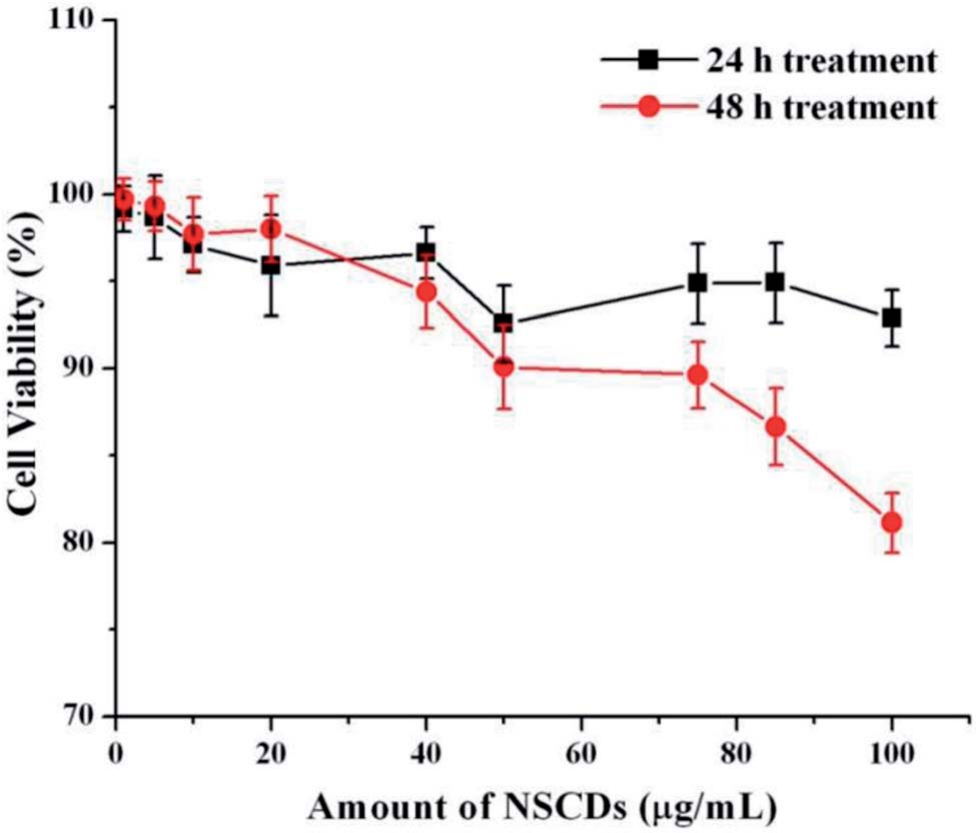
The effect of NSCDs of varying dose on growth of fibroblast cells after 24 h and 48 h treatment respectively measured by MTT assay.

We further evaluated the cytotoxic effect of NSCDs on human fibroblast cells using MTT assay. Human fibroblast cells were treated for 24 and 48 hours with different concentration of NSCDs to assess the cell viability. As shown in Fig. 10, the cell viability were estimated to be greater than 90% after 24 h incubation and 80% after 48 h of incubation upon addition of the as-prepared NSCDs within the concentration range of 0–100 μg mL^−1^. Thus, the cellular uptake study and MTT assay confirmed that the as-prepared NSCDs possessed negligible toxicity and excellent biocompatibility to normal human cells and could serve in bioimaging and various biomedical applications.

## 4. Conclusions

Fast and simple one-pot microwave-assisted pyrolysis route has been established to synthesize highly fluorescent N and S co-doped CDs (NSCDs) using thioacetamide as the source of N and S, tartaric acid as the source of carbon. The as-prepared NSCDs were spherical in shape with an average diameter of around 5 nm and emit bright blue photoluminescence with a quantum yield of ∼12%. The NSCDs presented various merits including excitation wavelength dependent emission, excellent photostability, low cytotoxicity and satisfactory aqueous solubility. Moreover, NSCDs show superior sensitivity and selectivity to cinnamaldehyde over various aliphatic and aromatic aldehydes and thus NSCDs can be served as an efficient fluorescent probe for detecting cinnamaldehyde in the linear range of 0–15 μM with a detection limit of 99.0 μM. The fluorescence quenching of NSCDs with the addition of cinnamaldehyde is mainly achieved by photo-excited electron transfer in between them and the process is thermodynamically feasible with Δ*G* value of −38.05 kcal mol^−1^. The NSCDs-based simple, low-cost and convenient fluorescent probe provides a new platform for the detection of cinnamaldehyde and synthesized NSCDs may extend their great potential in cell imaging and drug delivery applications.

## Conflicts of interest

There are no conflicts to declare.

## Supplementary Material

RA-008-C8RA09285K-s001
