# Supplementary material for: Selective and sensitive detection of cinnamaldehyde by nitrogen and sulphur co-doped carbon dots: a detailed systematic study
Source: RSC Adv. 2018 Dec 19;8(74):42361–73. doi: 10.1039/c8ra09285k (PMC9092083; doi:10.1039/c8ra09285k)
Supplement: RA-008-C8RA09285K-s001 [file RA-008-C8RA09285K-s001.pdf]

Electronic Supplementary Material (ESI) for RSC Advances.
This journal is © The Royal Society of Chemistry 2018

Supplementary Information

Selective and Sensitive Detection of Cinnamaldehyde by Nitrogen and Sulphur co-doped Carbon Dot: A Detail Systematic Study

Suraj Konar,[a,b] Dipanjan Samanta,[a] Subhajit Mandal,[c] Subhayan Das,[d] Madhusudan Kr Mahto,[a] Manisha Shaw[a], Mahitosh Mandal,[d] Amita Pathak*,[a]

[a] Department of Chemistry, Indian Institute of Technology, Kharagpur, West Bengal, 721302, India

[b]Department of Chemistry, R.D. & D.J. College Munger, Munger University, Bihar, 811201, India

[c]Raman Center for Atomic, Molecular and Optical Science, Indian Association for the Cultivation of Science, Kolkata, West Bengal, 700032, India

[d] School of Medical Science and Technology, Indian Institute of Technology, Kharagpur, West Bengal, 721302, India

1. Quantum yield (QY) measurements:

The quantum yield of NSCDs was determined with respect to quinine sulphate (Sigma-Aldrich, Germany, 99%) dissolving 0.1 M H_2SO_4 (quantum yield 54%) as standard [39]. The absorbances of various NSCDs solutions and quinine sulphate solutions were measured in UV-Visible

spectrophotometer (SHIMADZU UV-2450, Japan). The quantum yield of NSCDs was calculated by using the following equation:

$$\frac{\emptyset}{\emptyset_{Std}} = \frac{A_{Std}}{A} \times \frac{I}{I_{Std}} \times \frac{n^2}{n_{Std}^2}$$

Where $\emptyset$ stands for quantum yield, I stands for measured integrated fluorescence intensity, n is the refractive index and A is the absorbance. "Std" refers to the standard fluorophore (quinine sulphate). The absorbance of all the solutions was kept less than 0.1 to avoid inner filter effects.

To find out the QY we have plotted integrated fluorescence intensities versus absorbances of various NSCDs and standard solutions and the following equation was used:

$$\emptyset = \emptyset_{Std} \times \frac{Slope}{Slope_{Std}} \times \frac{n^2}{n_{Std}^2}$$

where "Slope" represents the slope of the plot of absorbance versus integrated intensity [39].

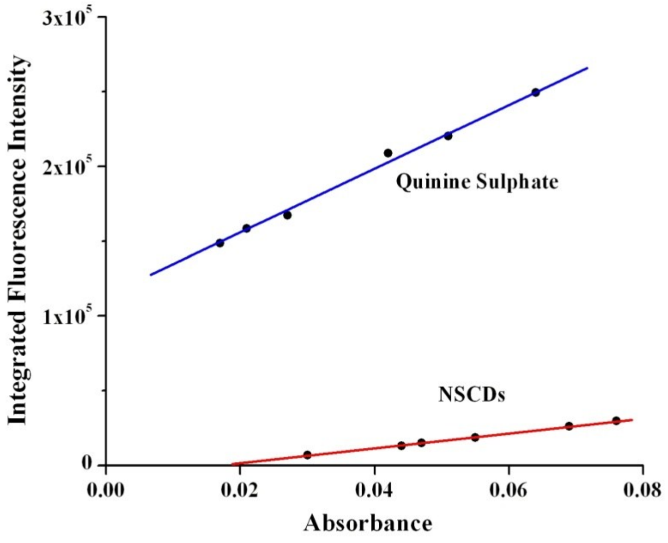

Fig. S1 Quantum yield measurement of NSCDs with reference to quinine sulphate in 0.1 M H_2SO_4.

2. Evaluation of TCSPC data:

Table S1. Fluorescence lifetime decay of the NSCDs solution in absence and presence of cinnamaldehyde.

**Sample**	**τ_1 (ns)**	**a_1**	**τ_2 (ns)**	**a_2**	**τ_3 (ns)**	**a_3**	**τ_{av} (ns)**
NSCDs	2.21	0.34	9.22	0.17	0.29	0.49	2.44
NSCDs + 8mM cinnamaldehyde	2.23	0.33	9.04	0.16	0.29	0.51	2.36
NSCDs + 16mM cinnamaldehyde	2.15	0.31	8.74	0.16	0.25	0.53	2.19
NSCDs + 24mM cinnamaldehyde	2.001	0.27	8.12	0.14	0.20	0.59	1.814

***Chi square: 1.1**

Multi-component fit to the excited state lifetime decay kinetics of the NSCDs in presence and absence of cinnamaldehyde suggests the presence of multiple radiative species in the samples. We considered the average lifetimes of both the species. The average lifetimes (τ_{av}) of the NSCDs in presence and absence of cinnamaldehyde were calculated using the equation: $\tau_{av} = (a_1\tau_1 + a_2\tau_2 + a_3\tau_3)/(a_1 + a_2 + a_3)$, where τ_1, $\tau_2$and $\tau_3$are the fluorescence lifetimes (in ns) and a_1, $a_2$and $a_3$are their relative amplitudes.

3. Cyclic voltammogram analysis of NSCDs:

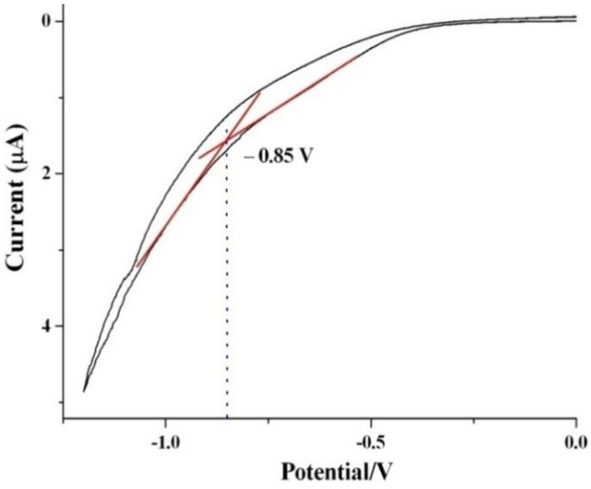

Fig.S2 Cyclic voltammogram curve of the NSCDs. The sample was prepared by dip-coating the NSCDs solution on glass carbon electrode.

4. Density Functional Theory (DFT) analysis:

All the theoretical calculations were performed using the Gaussian 09 software package (revision D.01).[T1]

T1. M. J. Frisch, G. W. Trucks, H. B. Schlegel, G. E. Scuseria, M. A. Robb, J. R. Cheeseman, G. Scalmani, V. Barone, B. Mennucci, G. A. Petersson, H. Nakatsuji, M. Caricato, X. Li, H. P. Hratchian, A. F. Izmaylov, J. Bloino, G. Zheng, J. L. Sonnenberg, M. Hada, M. Ehara, K. Toyota, R. Fukuda, J. Hasegawa, M. Ishida, T. Nakajima, Y. Honda, O. Kitao, H. Nakai, T. Vreven, J. A. Montgomery, Jr., J. E. Peralta, F. Ogliaro, M. Bearpark, J. J. Heyd, E. Brothers, K. N. Kudin, V. N. Staroverov, T. Keith, R. Kobayashi, J. Normand, K. Raghavachari, A. Rendell, J. C. Burant, S. S. Iyengar, J. Tomasi, M. Cossi, N. Rega, J. M. Millam, M. Klene, J. E. Knox, J. B. Cross, V. Bakken, C. Adamo, J. Jaramillo, R. Gomperts, R. E. Stratmann, O. Yazyev, A. J. Austin, R. Cammi, C. Pomelli, J. W. Ochterski, R. L. Martin, K. Morokuma, V. G. Zakrzewski, G. A. Voth, P. Salvador, J. J. Dannenberg, S. Dapprich, A. D. Daniels, O. Farkas, J. B. Foresman, J. V. Ortiz, J. Cioslowski, and D. J. Fox, Gaussian 09 (Revision D.01), Gaussian, Inc., Wallingford CT, 2013.

Table S2. Comparison of energy of highest occupied molecular orbital (E_H), energy of lowest unoccupied molecular orbital (E_L) and the energy gap between the LUMO and HOMO (E_g) at several DFT level of theory.

Aldehydes	**B3LYP/6–31+g(d)**			**ωB97X–D/6–31+g(d)**			**PBE0/6–31+g(d)**			**M06–2X/6–31+g(d)**		
	E_H (eV)	E_L (eV)	E_g (eV)	E_H (eV)	E_L (eV)	E_g (eV)	E_H (eV)	E_L (eV)	E_g (eV)	E_H (eV)	E_L (eV)	E_g (eV)
Formaldehyde	–11.18	–1.71	9.47	–13.45	0.50	13.95	–11.50	–1.37	10.13	–12.99	–0.32	12.67
Acetaldehyde	–10.23	–1.08	9.15	–12.42	1.12	13.54	–10.49	–0.75	9.74	–11.93	0.32	12.25
Propionaldehyde	–9.75	–0.98	8.77	–11.88	1.21	13.09	–9.94	–0.66	9.28	–11.32	0.38	11.70
Butyraldehyde	–9.55	–1.08	8.47	–11.45	1.22	12.67	–9.73	–0.76	8.97	–10.88	0.39	11.28
Valeraldehyde	–9.51	–1.06	8.45	–11.65	1.12	12.77	–9.77	–0.74	9.03	–10.87	0.40	11.27
Hexanal	–9.45	–0.96	8.49	–11.57	1.23	12.80	–9.63	–0.63	9.00	–11.01	0.40	11.41
Crotonaldehyde	–7.81	–1.88	5.93	–9.84	0.18	10.02	–8.03	–1.63	6.40	–9.22	–0.65	8.57
Benzaldehyde	–7.49	–2.13	5.36	–9.52	–0.18	9.34	–7.73	–1.92	5.81	–8.89	–1.04	7.85
Cinnamaldehyde	–6.89	–2.48	4.41	–8.77	–0.59	8.18	–7.11	–2.29	4.82	–8.14	–1.45	6.69

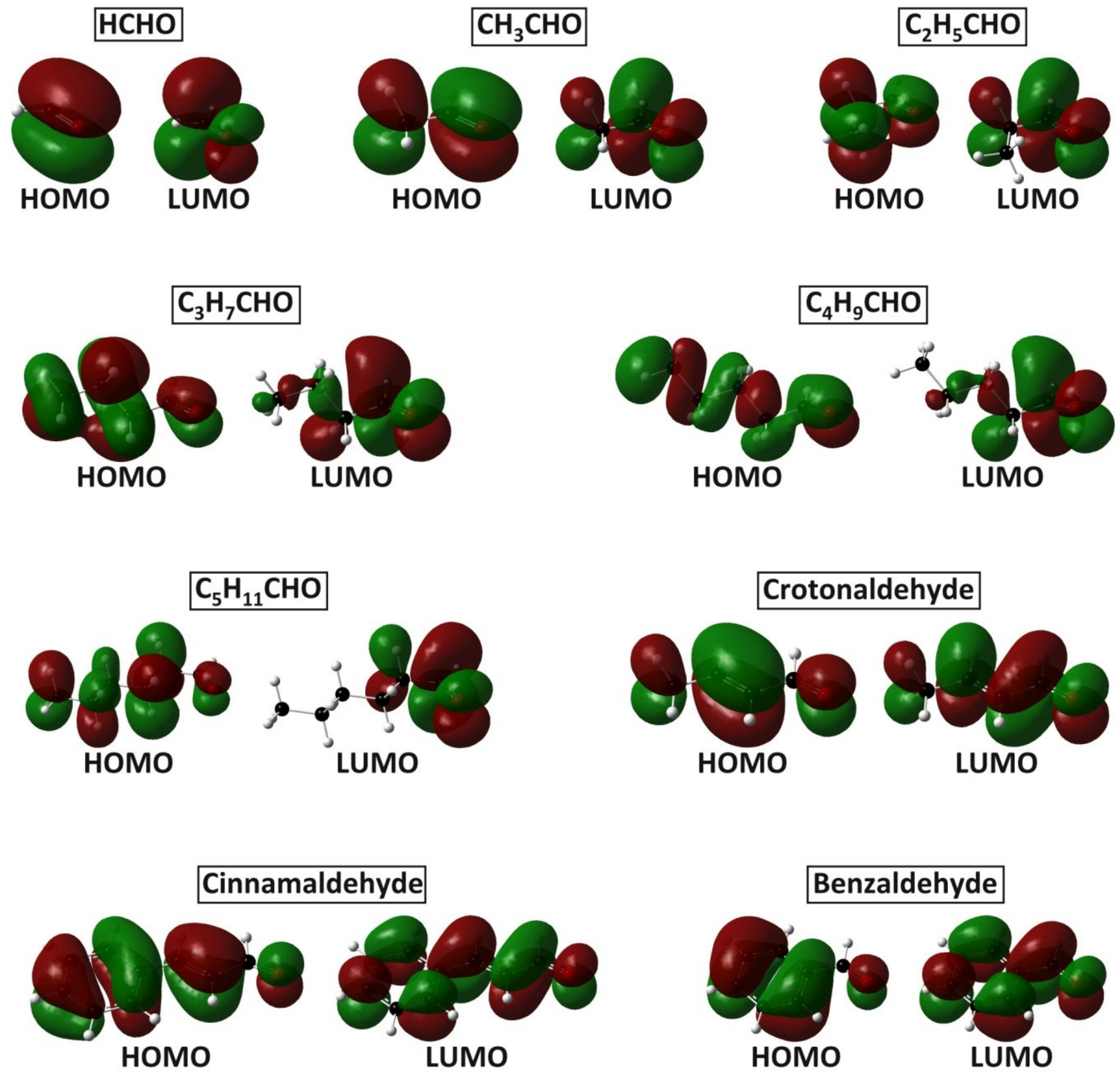

Fig.S3 Pictorial depiction of HOMOs and LUMOs of the studied aldehydes.
